# Multi-Run Concrete Autoencoder to Identify Prognostic lncRNAs for 12 Cancers

**DOI:** 10.3390/ijms222111919

**Published:** 2021-11-03

**Authors:** Abdullah Al Mamun, Raihanul Bari Tanvir, Masrur Sobhan, Kalai Mathee, Giri Narasimhan, Gregory E. Holt, Ananda Mohan Mondal

**Affiliations:** 1 Knight Foundation School of Computing and Information Sciences, Florida International University, Miami, FL 33199, USA; mmamu009@fiu.edu (A.A.M.); rtanv003@fiu.edu (R.B.T.); msobh002@fiu.edu (M.S.); giri@fiu.edu (G.N.); 2 Department of Human and Molecular Genetics, Herbert Wertheim College of Medicine, Florida International University, Miami, FL 33199, USA; matheek@fiu.edu; 3 Biomolecular Sciences Institute, Florida International University, Miami, FL 33199, USA; 4 Department of Medicine, Miami VA Healthcare System, Miami, FL 33125, USA; gholt@med.miami.edu; 5 Department of Medicine, University of Miami, Miami, FL 33146, USA

**Keywords:** autoencoder, concrete autoencoder, deep learning, feature selection, lncRNA, mrCAE

## Abstract

Background: Long non-coding RNA plays a vital role in changing the expression profiles of various target genes that lead to cancer development. Thus, identifying prognostic lncRNAs related to different cancers might help in developing cancer therapy. Method: To discover the critical lncRNAs that can identify the origin of different cancers, we propose the use of the state-of-the-art deep learning algorithm concrete autoencoder (CAE) in an unsupervised setting, which efficiently identifies a subset of the most informative features. However, CAE does not identify reproducible features in different runs due to its stochastic nature. We thus propose a multi-run CAE (mrCAE) to identify a stable set of features to address this issue. The assumption is that a feature appearing in multiple runs carries more meaningful information about the data under consideration. The genome-wide lncRNA expression profiles of 12 different types of cancers, with a total of 4768 samples available in The Cancer Genome Atlas (TCGA), were analyzed to discover the key lncRNAs. The lncRNAs identified by multiple runs of CAE were added to a final list of key lncRNAs that are capable of identifying 12 different cancers. Results: Our results showed that mrCAE performs better in feature selection than single-run CAE, standard autoencoder (AE), and other state-of-the-art feature selection techniques. This study revealed a set of top-ranking 128 lncRNAs that could identify the origin of 12 different cancers with an accuracy of 95%. Survival analysis showed that 76 of 128 lncRNAs have the prognostic capability to differentiate high- and low-risk groups of patients with different cancers. Conclusion: The proposed mrCAE, which selects actual features, outperformed the AE even though it selects the latent or pseudo-features. By selecting actual features instead of pseudo-features, mrCAE can be valuable for precision medicine. The identified prognostic lncRNAs can be further studied to develop therapies for different cancers.

## 1. Introduction

Recent studies have shown that long non-coding RNAs (lncRNAs), which are longer than 200 nucleotides, play key roles in tumorigenesis [1,2,3]. lncRNAs also have key functions in transcriptional, post-transcriptional, and epigenetic gene regulation [4]. Schmitt and Chang discussed the impact of lncRNA in cancer pathways [5]. Hanahan and Weinberg described the involvement of lncRNAs in six hallmarks of cancer such as proliferation, growth suppression, motility, immortality, angiogenesis, and viability [6].

Hoadley et al. showed that cell-of-origin patterns dominate the molecular classification of tumors available in The Cancer Genome Atlas (TCGA) [7]. Their analysis used copy number, mutation, DNA methylation, RPPA protein, mRNA, and miRNA expression. However, they did not consider another important molecular signature of cancer: lncRNA expression. This work motivated us to investigate the importance of lncRNAs in identifying different types of cancer. We hypothesized that there should be a shortlist of salient features or important lncRNAs with prognostic capability that could dictate the origin of multiple cancers.

In general, feature selection is worthwhile when the whole set of features is difficult to collect or expensive to generate [8]. For example, in TCGA, the lncRNA expression profile dataset contains more than 12,000 features (lncRNAs) for 33 different cancers, and it is expensive to generate these data.

Standard dimension reduction methods, such as principal component analysis (PCA) [9] and autoencoders [10], can generate a greatly reduced set of latent features. However, these latent features are not the original features but functional combinations of the original features. Identifying original features increases the explainability of results and allows one to perform biological interpretation when diagnosing various deadly diseases, such as cancers. Recently, a few deep learning-based feature selection methods showed improvement in selecting original features in both supervised and unsupervised settings [8,11,12,13].

In our previous study [14], we showed that a deep learning-based unsupervised feature selection algorithm CAE [8] performed better in feature selection, especially in selecting a small number of features, compared to state-of-the-art supervised feature selection methods such as least absolute shrinkage and selection operator (LASSO) [15], random forest (RF) [16], and support vector machine with recursive feature elimination (SVM–RFE) [17]. However, the study was only based on the expression profiles of cancer patients. Questions remained unanswered regarding (a) whether the identified lncRNAs were cancer-specific or organ-specific, (b) which set to use as the final feature set given that CAE produces a different set of features in different runs, (c) whether the identified lncRNAs have prognostic capability, and (d) the validation method of the identified lncRNAs.

In this paper, to address question (a), we analyzed data from 12 cancers, with a normal to cancer sample ratio of at least 1:10. To address question (b), we ran CAE multiple times, with a fixed number of features to be selected in each run and the most frequently appearing features in multiple runs taken as the final set of features. To address question (c), survival analysis was performed to show that the identified features have prognostic capability. To address question (d), we checked the existence of identified lncRNAs in experimental works of literature, drug–lncRNA networks, and cancer hallmarks.

The contributions of this study are as follows: (1) the development of an optimal and stable feature selection framework, mrCAE; (2) the discovery of an optimal and stable set of 128 lncRNAs capable of identifying the origin of organs for 12 different cancers with an accuracy of 95%; (3) the demonstration that the lncRNAs identified using mrCAE from the expression profiles of cancer patients are truly cancer-specific, not organ-specific; (4) the survival or prognostic analysis of discovered lncRNAs; and (5) the validation of identified features, lncRNAs, with existing literature, drug–lncRNA networks, and hallmark lncRNAs.

## 2. Results

The lncRNA expression profiles of 12 cancers were analyzed with the goal of identifying the key lncRNAs using mrCAE. First, we showed that the features selected by CAE were truly cancer-specific, not organ-specific. Second, we showed the stochastic nature of CAE in selecting equally significant different sets of features in different runs. Third, we showed that mrCAE performed better than the single-run CAE and other state-of-the-art feature selection methods (LASSO, RF, SVM–RFE, MCFS, and UDFS). Fourth, we determined a stable set of lncRNAs that not only could stratify 12 different cancer types but also had the highest number of lncRNAs with prognostic behavior.

### 2.1. Features Selected from Tumor Tissues Are Cancer-Specific Not Organ-Specific

**Figure 1:**
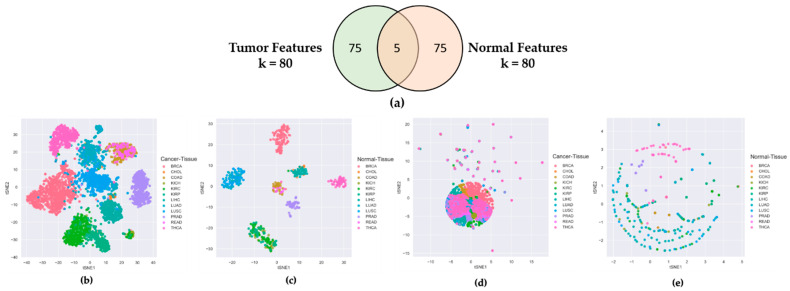
Comparing Tumor Features with Normal Features. (**a**) Venn diagram of 80 tumor features and 80 normal features derived from CAE; (**b**) t-SNE plot of tumor samples using tumor features; (**c**) t-SNE plot of normal samples using normal features; (**d**) t-SNE plot of tumor samples using normal features; (**e**) t-SNE plot of normal samples using tumor features.

To check that the features selected by CAE from the lncRNA expression profiles of cancer samples were truly cancer-specific, not organ-specific, we separately ran CAE on tumor and normal samples to identify two sets of 80 features (lncRNAs). Figure 1a shows only five commons between 80 tumor and 80 normal features, which evidenced that 75 out of 80 features were unique to both tumor and normal tissues. It is clear from the t-SNE plots of Figure 1b,c that the tumor and normal features could distinctively cluster 12 tumor tissues and corresponding normal tissues, respectively. However, when we cross-validated the t-SNE plot of tumor tissues using normal features (Figure 1d) and the t-SNE plot of normal tissues using tumor features Figure 1e, we found no distinct clusters for 12 tumor and corresponding normal tissues. *Supplementary 2* shows similar results for the 40-feature and 60-feature scenarios. These experiments proved that the features derived from tumor samples were truly cancer-specific, not organ-specific.

### 2.2. CAE Produces Different Sets of Significant Features in Different Runs

**Figure 2:**
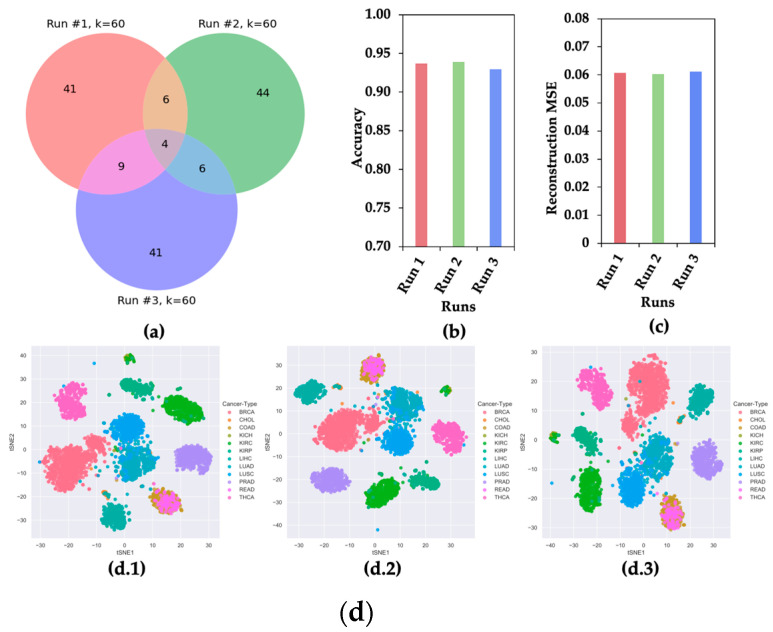
**CAE Property of Selecting Different Sets of Features in Different Runs.** (**a**) Venn Diagram, (**b**) accuracy of classifying 12 cancer types, (**c**) reconstruction mean squared error (MSE), and (**d**) t-SNE plots for 12 cancer samples using three sets of 60 features selected in three runs.

Though CAE selects a subset of the most significant features from a given dataset, it produces different sets of significant features in different runs due to its stochastic nature [8]. To show the stochastic nature of CAE, three sets of 60 features were selected for the experiment. Figure 2 shows the (a) Venn diagram, (b) classification accuracy of 12 cancer types, (c) mean squared error (MSE) of reconstructing original feature space, and (d) t-SNE plots of clustering 12 different types of cancer samples using three sets of features.

Figure 2 presents evidence that the CAE selected different sets of most informative features in different runs. This observation motivated us to hypothesize that a feature appearing in multiple runs of CAE (mrCAE) carries the most meaningful information for a given dataset.

### 2.3. Comparison of mrCAE with Existing Feature Selection Approaches

**Figure 3:**
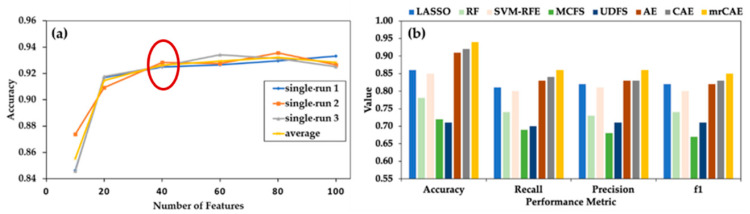
**Comparing mrCAE with other feature selection approaches.** (**a**) Behavior of single-run CAE to decide the number of features to be selected for comparison. CAE was run three times to select six sets of 10, 20, 40, 60, 80, and 100 features. “single avg” represents the average accuracy of three runs. (**b**) Classification performance using 40 features selected by LASSO, RF, SVM-RFE, MCFS, UDFS, AE, CAE and mrCAE. Note that, each approach selects 40 actual features except AE, which selectes 40 latent features.

Before comparing mrCAE with the existing feature selection approaches, we evaluated the performance of a single-run CAE with a different number of selected features, which guided us regarding how many features we had to select for comparison. In Figure 3a, it is noticeable that even with a smaller number of only ten features, the average accuracy of CAE was close to 85%. There was a sharp increase in average accuracy (91%) with 20 features, followed by a slight increase (92% accuracy) with up to 60 features. Then, the curve reached a plateau. This figure suggests that selecting 40 features (before starting plateau) while using different algorithms is a good choice for comparison.

Selection of 40 Features from mrCAE: CAE was run 100 times to select 100 features in each run. Over 100 runs, it selected a total of 534 unique features. The frequency of appearing these features in 100 runs ranged between 1 and 98. The 40 most frequent features, the top 40 features from the sorted list in descending order based on frequency, were used to measure the performance of mrCAE.

Figure 3b shows the classification performance when using the sets of 40 lncRNAs selected from the LASSO, RF, SVM–RFE, MCFS, UDFS, AE, CAE, and mrCAE feature selection algorithms. It is clear that mrCAE performed better than any other feature selection approaches in accuracy, recall, precision, and F1 score.

### 2.4. mrCAE to Select a Stable Set of Features

mrCAE Systems: To identify a unique and stable set of lncRNAs that not only can distinguish between 12 different cancer types but also have the highest number of features with prognostic behavior, we designed mrCAE systems with 10, 20, 40, 60, 80, 100, and 120 runs. In each of the single runs of an mrCAE system, 100 lncRNAs were selected. Table 1 shows the summary statistics of mrCAE systems, including the total number of unique lncRNAs selected and the maximum frequency of an lncRNA appearing in each mrCAE system. The minimum frequency was 1 for all the different mrCAE systems. As shown in Table 1, a total of 223 unique lncRNAs (combined list of 10 sets of 100 lncRNAs) were selected by the 10-run mrCAE system, and the frequency of an lncRNA appearing in multiple runs ranged between 1 and 10. Similarly, a total of 575 unique lncRNAs were selected by the 120-run mrCAE system, and the frequency of an lncRNA appearing in multiple runs ranged between 1 and 117.

Frequent and Stable Features: Features appearing more than once in mrCAE system were considered frequent features. Features with higher frequencies were considered stable features.

The Top Frequent Features: The top frequent features, for example, Top-10 features in any mrCAE system, were the first ten features from the combined list sorted in descending order based on frequency. To identify a stable set of lncRNAs, we selected the top features from each of the seven mrCAE systems in six different categories: Top-10, Top-20, Top-40, Top-60, Top-80, and Top-100. Table 2 shows the ranges of frequency for the top features in six different categories. It is noticeable that the most frequent feature appeared in 10, 20, 40, 60, and 80 runs in the cases of 10-, 20-, 40-, 60- and 80-run mrCAE systems, respectively, but the trend was not maintained for 100- and 120-run systems. In other words, the most frequent feature appeared in each run of each mrCAE system except for the 100-run and 120-run systems, for which it (most frequent feature) appeared in 98 and 117 runs, respectively. It can be concluded that for the given lncRNA expression profile dataset of 12 cancers, the mrCAE system with 100 or more runs could not produce the most frequent features in each run. Thus, a 100-run mrCAE can be considered to be the optimal configuration for this dataset, and the results from 120-run mrCAE were not considered for subsequent analyses.

**Figure 4:**
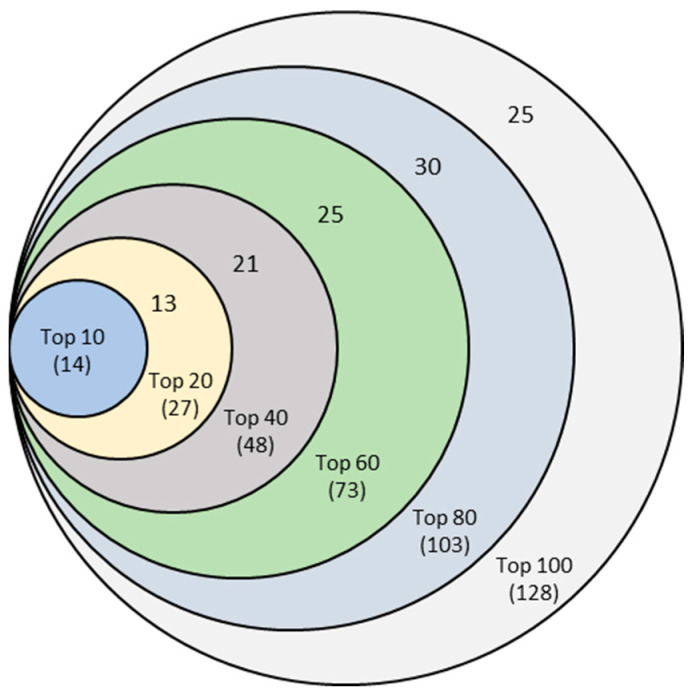
Venn diagram of six sets of unique features identified from six mrCAE systems. The mrCAE consisted of 10, 20, 40, 60, 80, and 100 runs. Each of these runs was conducted to select 100 features. The smallest set (light blue), containing 14 features, represents the unique features coming from six sets of 10 most frequent features from 10-, 20-, 40-, 60-, 80-, and 100-run mrCAE systems. Similarly and so on, the 2nd smallest set contains 27 (14 + 13) unique features from six sets of Top-20 features selected.

Finally, this experiment resulted in six unique sets of features corresponding to Top-10, Top-20, Top-40, Top-60, Top-80, and Top-100 features, as shown in the Venn diagram of Figure 4. For example, combining six sets of top-10 features from 10-, 20-, 40-, 60-, 80-, and 100-run mrCAE systems produced a unique list of 14 lncRNAs.

The Venn diagram shows that each set of unique features was a subset of the following more extensive unique feature set. Finally, we can conclude that the 128 unique features (*Supplementary 3*)—produced from the union of six sets of Top-100 features coming from 10-, 20-, 40-, 60-, 80-, and 100-run mrCAE systems—represented the stable and optimal feature set. We used this set of lncRNAs to conduct the downstream study, including survival and prognostic analyses and validation.

### 2.5. Prognostic Capability of Significant lncRNAs

To evaluate the prognostic capabilities of the selected 128 stable lncRNAs, survival analyses of patients with different cancer types were performed. Any lncRNAs with zero expression values for most of the cancer samples were excluded from the survival analysis of that cancer. The patients with values less than or equal to the median were labeled group A. Those with values greater than the median were labeled group B. After dividing into two groups, a log-rank test was conducted, and the hazard ratio was calculated as the hazard rate of group A vs. hazard rate of group B to check the prognostic capability of an lncRNA. The criteria for an lncRNA to be prognostic are log-rank test *p*-value ≤ 0.05 and Hazard Ratio (HR) ≠ 1.0. Kaplan–Meier curves were plotted to show the prognostic behavior of lncRNAs.

**Figure 5:**
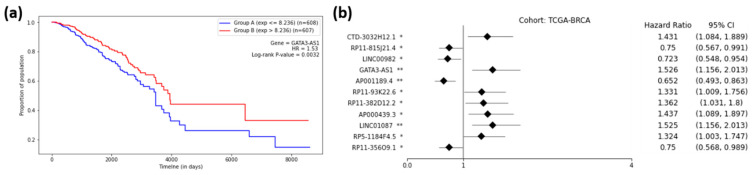
**Survival Analysis of TCGA-BRCA.** (**a**) Kaplan–Meier Curve for the GATA3-AS1 lncRNA on the TCGA-BRCA cohort. Group A (blue) is the group with an expression less than or equal to the median, and Group B (red) is the group with an expression greater than the median. (**b**) Forest plot of survival analysis for 11 prognostic lncRNAs on the BRCA cohort. The asterisks represent the log-rank *p*-values (*—*p* ≤ 0.05, **—*p* ≤ 0.01).

Figure 5a shows the Kaplan–Meier plot for GATA3-AS1, one of the 11 prognostic lncRNAs for breast cancer, and Figure 5b shows the forest plot of survival analyses for 11 prognostic lncRNAs. It can be observed from Figure 5a that group B (red) had a higher rate of survival than group A (blue), meaning that lncRNA GATA3-AS1 could successfully distinguish the high-risk group (Group A) of BRCA patients from the low-risk group (Group B). In other words, the cohort with a low expression (blue) of GATA3-AS1 had a 1.53-times higher rate of death than the high-expression cohort (red). Thus, the cohorts with low-expression values for seven lncRNAs (HR > 1.0) showed higher chances of death compared to the high-expression cohorts (Figure 5b). On the other hand, the cohorts with low-expression values for four lncRNAs (HR < 1.0) showed lower chances of death compared to the high-expression cohorts (Figure 5b). *Supplementary 4* shows the forest plots for other cancer types.

The number of prognostically significant lncRNAs for each type of cancer is given in Table 3. The highest number of prognostic lncRNAs were discovered for KIRC (31 lncRNAs), followed by LUAD (22 lncRNAs) and LUSC (18 lncRNAs). The proposed approach failed to discover any prognostic lncRNA for CHOL, potentially because the cohort consisted of only 36 patients (Table 1). Some of the lncRNAs were found to be prognostic for more than one cancer. Of 128 stable set of lncRNAs, 76 were found to be prognostic.

### 2.6. Validations

**Figure 6:**
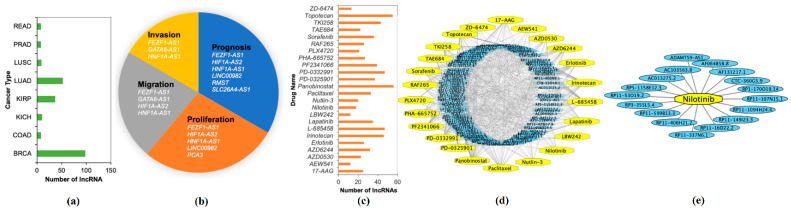
**Validation of Identified lncRNAs.** (**a**) Number of known lncRNAs derived by mrCAE related to different cancer types found in [20,21,22,23]; (**b**) mrCAE derived lncRNAs related to different cancer hallmarks [24]; (**c**) number of lncRNAs, related to different cancer drugs [19]; (**d**) drug–lncRNA networks for all 24 drugs; (**e**) an example lncRNA–drug network for nilotinib, which is used to treat certain blood cancers associated with 18 different lncRNAs. (**d**,**e**) were generated using Cytoscape.

The stable set of 128 lncRNAs derived from mrCAE was validated with the existing literature [18]. Of 128 lncRNAs, 103 were found to be known lncRNAs (*Supplementary 5*) associated with different cancer types; see Figure 6a. For example, 98 lncRNAs are associated with BRCA, 52 lncRNAs are related to LUAD, and 37 lncRNAs are related to KIRP. Some lncRNAs were also found in four different cancer hallmarks (Figure 6b and *Supplementary 6*); for example, six lncRNAs were found to be related to cancer prognosis. We also validated the top 128 lncRNAs with existing drug–lncRNA networks (*Supplementary 7*). We found that 113 out of 128 lncRNAs are associated with 24 different drugs primarily used in cancer-related treatments, as shown in Figure 6c,d. For example, the drug nilotinib is mainly used to treat a specific type of blood cancer associated with 18 different lncRNAs (Figure 6e); a drug–lncRNA network was formed based on the Spearman correlation coefficient between lncRNA expression levels and the IC50 values of the drug [19].

## 3. Discussion

The objective of the present study was to identify significant lncRNAs that carry meaningful information on (a) identifying the origins of multiple cancer, (b) evaluating the prognostic capability of differentiating high-risk and low-risk groups of patients of particular cancers, and (c) having potential for targeted therapy. The original CAE algorithm is capable of identifying subsets of important features. However, due to the stochastic nature of the algorithm, it produces different subsets in different runs [8]. Thus, our hypothesis was that the most frequently appearing lncRNAs in multiple runs of CAE (mrCAE) would produce a biologically meaningful set of features.

Our investigation showed that the lncRNAs selected by the proposed mrCAE carry meaningful information on the prognostic capability of differentiating high- and low-risk groups of patients of particular cancers, as explained in Section 2.5. We also showed the biological relevance of the selected lncRNAs by comparing them with existing literature, drug–lncRNA networks, and hallmark lncRNAs (Figure 6).

Figure 5 shows that the lncRNAs selected by the proposed mrCAE outperformed both the single-run CAE and the standard autoencoder, along with other feature selection approaches. Thus, the current results confirmed that the proposed mrCAE could be utilized as a tool for identifying a stable set of meaningful features. It should be noted that the proposed mrCAE approach is very similar to a common bioinformatic approach of bootstrapping analysis used to evaluate the stability of results. A shortcoming of CAE is that it produces different sets of the most informative features in different runs, which makes it difficult to use in precision medicine. We propose using a multi-run CAE approach to reduce the stochasticity in CAE outcomes, i.e., to select a stable set of features. The frequent features that appear in multiple runs are considered to be the stable set of features. The bootstrapping effect could be the reason that mrCAE performs better than the CAE and standard AE.

## 4. Materials and Methods

### 4.1. Data Preparation

To characterize the cancer-associated lncRNA, expression profiles and clinical data for 33 different cancers were downloaded from the UCSC Xena database [25]. Each lncRNA expression was processed using a min–max normalization method to achieve good training performance. For this study, we considered the cancer types for which the number of normal samples was at least 10% of cancer samples, and 12 cancer types met this criterion. The distributions of cancer and normal samples for 12 cancers are shown in Table 4.

This dataset contained about 60 thousand RNAs expression profiles, including coding genes (mRNAs) and non-coding genes (lncRNAs and miRNAs). In this study, only the expression profiles of lncRNA (n = 12,309) were considered for analysis and model evaluation. The final dataset contained 4768 cancer patients and 565 normal patients.

### 4.2. Features Selection Using Multi-Run Concrete Autoencoder

To select important features (lncRNAs), a state-of-the-art deep learning-based unsupervised algorithm, concrete autoencoder (CAE) [8], was iteratively run multiple times. We named this approach multi-run CAE (mrCAE). The reason for using mrCAE is that CAE selects the most informative features in a stochastic manner, meaning that different sets of informative features are selected in different runs. The assumption we made while running CAE multiple times was that if a feature appeared in more than one run, it can be considered a stable feature.

#### 4.2.1. Architecture and Working Principle of CAE

**Figure 7:**
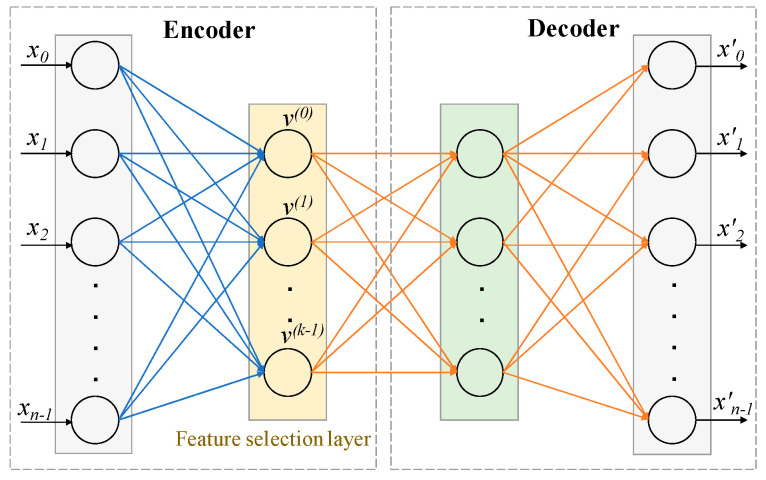
**Architecture of Concrete Autoencoder**. CAE architecture consists of an encoder and a decoder. The layer after the input layer of the encoder is called the concrete feature selection layer, as shown in yellow. This layer has k number of nodes, where each node is used for each feature to be selected. The decoder is used to check how well the input features can be reconstructed using the selected *k* features. The output layer has the same number of nodes as the input layer. *X* = *[x*_0_, *x*_1_, …., *x*_*n*−1_*]* = input features. *X*^′^ = *[x*^′^_0_, *x*^′^_1_, …., *x*^′^_*n*−1_*]* = reconstructed features.

The architecture of the CAE shown in Figure 7 consisted of a single encoding layer, also known as the feature selection layer shown in yellow, and arbitrary decoding layers (e.g., a deep feedforward neural network), shown in the box on the right. The detailed algorithm is available in [8]. The function of the encoder is to select a given number of k actual features (not latent features in the case of a traditional Autoencoder) in a stochastic manner from the original large input feature space X of size n. The function of the decoder is to reconstruct the original features ($X^{\prime }$ is the reconstructed feature vector) using the k features selected by the encoder.

How input features are selected depends on the temperature of the selection layer, which is modulated from a high value to a small value using a simple annealing schedule [8]. As the temperature of the selection layer approaches zero, the layer selects *k* individual input features. The decoder of a concrete autoencoder serves as the reconstruction function. It is the same as that of a standard autoencoder. Thus, the concrete autoencoder can be used to select a discrete set of *k* features that are optimized for an arbitrarily complex reconstruction function.

Training and Testing/Validation of CAE: The samples in a cohort were divided into 80/20 split in a stratified manner for training and testing. In the training phase, 80% of samples were used to select the *k* informative features. In the testing/validation phase, 20% of samples were used to reconstruct their original features using the selected *k* features.

#### 4.2.2. Hyperparameter Tuning for CAE

The hyperparameters of CAE were tuned for the lncRNA expression data of 12 TCGA cancer types. We kept two of the parameters the same as those used in the original CAE developed by Abid et al. [8]. These two parameters were leaky ReLU with a threshold value of 0.1 and a 10% dropout rate. To tune the number of nodes in two hidden layers of the decoder, the model was tested by varying the number of nodes from 240 to 340 with a step size of 10. It was found that a decoder with 300 nodes in both layers yielded the highest accuracy. Thus, the number of nodes in two hidden layers of the decoder was selected to be 300.

To tune the number of epochs and learning rate, the random search [26] approach was used. For the number of epochs, we used values if 200, 300, 500, 1000, 1500, 2000, 2500, and 3000. Similarly, for the learning rate, the values were 0.001, 0.002, 0.005, 0.0005, 0.01, and 0.05. In every run of CAE, the values of the two hyperparameters were randomly selected. With 300 epochs and 0.002 learning rate, the 100 features selected by the CAE produced the highest accuracy in classifying 12 cancer types using SVM. So, these parameter values were chosen for further analysis. Details of hyperparameter tuning are available in *Supplementary 1*.

**Figure 8:**
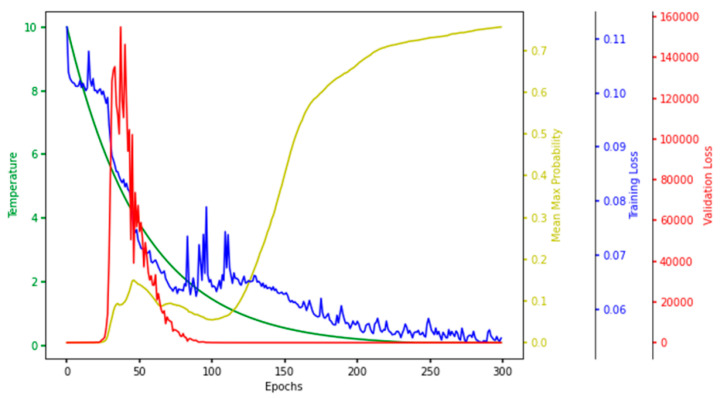
**Characteristic Plot of Concrete Autoencoder.** Temperature (green), mean–max probability (yellow), training loss (blue), and validation loss (red) are plotted at different scales.

For every iteration of a single run in the hyperparameter tuning phase, the temperature, mean–max probability (mean of maximum probabilities of the selected features), training loss, and validation loss were observed and plotted. The plot painted a clear picture of the learning process in the CAE at every epoch, so we named it the characteristic plot of CAE and present it in Figure 8.

One of the main objectives of making this plot was to see if the model converged in terms of loss, which is evident in Figure 8, which shows that the training and validation loss converged to a lower value. Each node in the concrete selection layer learned a probability value for every feature, and the node selected the one with the highest probability. The higher the mean–max probability was, the more each node in the concrete selector was confident of one of the features. So, the mean–max probability should be as high as possible.

### 4.3. Comparing mrCAE with Other Feature Selection Approaches

The feature selection capability of the mrCAE was compared with the standard autoencoder (AE), three frequently used embedded feature selection models (LASSO [15], random forest (RF) [16], and support vector machine with recursive feature elimination (SVM–RFE) [17]), and two unsupervised feature selection models (multi-cluster feature selection (MCFS) [27] and unsupervised discriminative feature selection (UDFS) [28]). The same numbers of features were selected using all feature selection approaches for comparison, and those features were used to evaluate the classification performance in classifying 12 different cancer types. A stratified 5-fold cross-validation using SVM with linear kernel was conducted to evaluate the classification performance. Four different evaluation metrics—accuracy, precision, recall, and f1 score—were used to record the classification performance.

### 4.4. Implementation of Feature Selection Algorithms

All feature selection algorithm except for mrCAE were implemented using the scikit-learn framework (https://scikit-learn.org/ Accessed: Jun’20), whereas mrCAE was implemented using a deep learning framework named Keras (https://keras.io/ Accessed: Jun’20). Experiments were parallelized on NVIDIA Quadro K620 GPU with 384 cores and 2 GB memory devices. The dataset was split into the training and testing set according in an 80/20 ratio to avoid overfitting. The training set was used to estimate the learning parameters, and the testing set was used for performance evaluation.

## 5. Conclusions

The authors of this study proposed a multi-run concrete autoencoder (mrCAE) to identify prognostic lncRNAs for multiple cancers. We tested the proposed model in analyzing the lncRNA expression profiles of 12 cancers. The model selected a stable set of lncRNAs that could differentiate 12 cancers with high accuracy and provide subsets of prognostic lncRNAs for 12 cancers. Though the proposed mrCAE model was applied to multiple cancers here, it can also be used on a single cancer dataset, such as when it was used to identify informative features for single-digit MNIST data by the developer of CAE.

The lncRNAs selected by the proposed mrCAE outperformed the lncRNAs selected by the single-run CAE and other feature selection approaches. Additionally, the proposed mrCAE outperformed the standard autoencoder, which selected the latent features and was thought to be the upper limit in dimension reduction. Since the proposed mrCAE outperformed AE and can select actual features in contrast to latent features by AE, it can provide meaningful information that can be used for precision medicine, such as identifying prognostic lncRNAs for different cancers. The same approach can be used in identifying salient features in other omics data.

## Figures and Tables

**Table 1:** Summary statistics of mrCAE systems in selecting lncRNAs.

mrCAE	Total LncRNAs	Min Frequency	Max Frequency
10-run mrCAE	223	1	10
20-run mrCAE	313	1	20
40-run mrCAE	400	1	40
60-run mrCAE	464	1	60
80-run mrCAE	499	1	80
100-run mrCAE	534	1	98
120-run mrCAE	575	1	117

**Table 2:** Ranges of frequency for the top features in six categories.

	Ranges of Frequency					
mrCAE	Top-10	Top-20	Top-40	Top-60	Top-80	Top-100
10-run mrCAE	(10–10)	(9–10)	(6–10)	(4–10)	(3–10)	(2–10)
20-run mrCAE	(19–20)	(15–20)	(11–20)	(8–20)	(5–20)	(4–20)
40-run mrCAE	(36–40)	(29–40)	(22–40)	(15–40)	(11–40)	(8—40)
60-run mrCAE	(53–60)	(44–60)	(31–60)	(21–60)	(16–60)	(13–60)
80-run mrCAE	(69–80)	(60–80)	(42–80)	(28–80)	(22—80)	(17–80)
100-run mrCAE	(84–98)	(74–98)	(53–98)	(35–98)	(27–98)	(21–98)
120-run mrCAE	(99–117)	(85–117)	(62–117)	(44–117)	(34–117)	(25–117)

**Table 3:** Summary of survival analysis regarding the number of prognostic lncRNAs for each of the 12 TCGA cancer types.

BRCA	CHOL	COAD	KICH	KIRC	KIRP	LIHC	LUAD	LUSC	PRAD	READ	THCA	Total
11	0	3	3	31	15	1	22	18	4	4	10	**76**

**Table 4:** Sample distributions of 12 cancers considered in this experiment.

	BRCA	CHOL	COAD	KICH	KIRC	KIRP	LIHC	LUAD	LUSC	PRAD	READ	THCA
Normal	113	9	41	23	72	32	50	57	49	52	9	58
Cancer	1088	36	301	65	527	286	369	510	498	493	94	501

## Data Availability

The data used in this experiment were collected from *Xena browser*.

## Supplementary Materials

Supplemental Information (SI) is available at https://www.mdpi.com/article/10.3390/ijms222111919/s1.
