# Supplementary material for: Multi-Run Concrete Autoencoder to Identify Prognostic lncRNAs for 12 Cancers
Source: Int J Mol Sci. 2021 Nov 3;22(21):11919. doi: 10.3390/ijms222111919 (PMC8584911; doi:10.3390/ijms222111919)
Supplement: Supplementary file 1 [file ijms-22-11919-s001.zip › Supplementary/Supplementary_1_Hyperparameter Tuning.pdf]

Supplementary 1: Hyperparameter Tuning

In the decoder part of the CAE, different node values from 240 to 340, with a step size of 10 was tested to tune the number of nodes in the decoder layer. It was found the 300 nodes would yield the highest accuracy, as evident in Figure 1. So the number of nodes was selected as 300.

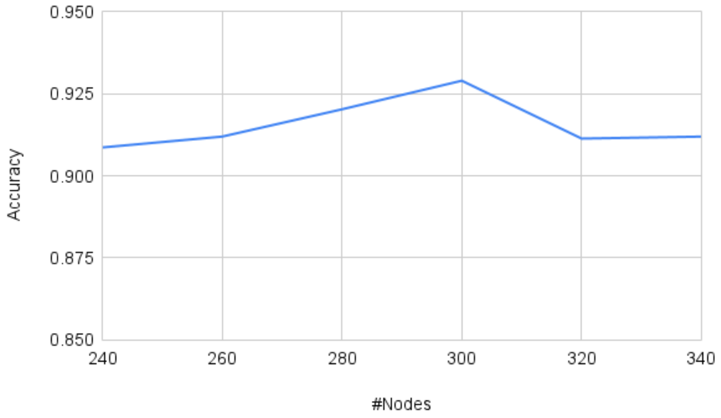

Figure 1. Tuning number of nodes in the decoder. For 300 nodes, it yields the highest accuracy.

We did a random search of parameters from a range of values to tune the number of epochs and learning rate. For the number of epochs, the used values are - 200, 300, 500, 1000, 1500, 2000, 2500, 3000. Similarly, for learning rate, the values are - 0.001, 0.002, 0.005, 0.0005, 0.01 and 0.05. Table 1 contains the accuracy of all different combinations of epoch and learning rate. The highest value of accuracy was 0.9507, which is found for the epoch of 300 and learning rate of 0.002.

Table 1. Summary of hyperparameter tuning for epoch and learning rates. For different values of epoch and learning rate, there are different accuracies for the SVM model and by the features selected by CAE.

Epoch	Learning Rate	Accuracy	Epoch	Learning Rate	Accuracy
200	0.0005	0.9256	500	0.005	0.9371
300	0.0005	0.9203	1000	0.005	0.9403
1000	0.0005	0.9340	1500	0.005	0.9266
1500	0.0005	0.9308	2000	0.005	0.9224
2000	0.0005	0.9277	2500	0.005	0.9308
2500	0.0005	0.9340	2500	0.005	0.9235
3000	0.0005	0.9434	3000	0.005	0.9224
200	0.001	0.9256	200	0.01	0.2296
300	0.001	0.9382	300	0.01	0.2180
500	0.001	0.9361	500	0.01	0.2317
1000	0.001	0.9444	1000	0.01	0.3071
1500	0.001	0.9476	1500	0.01	0.2453
2000	0.001	0.9340	2000	0.01	0.4130
2500	0.001	0.9497	2500	0.01	0.2233
3000	0.001	0.9413	3000	0.01	0.2914
200	0.002	0.9266	200	0.05	0.2421
300	0.002	0.9507	300	0.05	0.2411
500	0.002	0.9444	500	0.05	0.2254
1000	0.002	0.9486	1000	0.05	0.2222
1500	0.002	0.9392	1500	0.05	0.2379
2000	0.002	0.9434	2000	0.05	0.2254
2500	0.002	0.9319	2500	0.05	0.2285
3000	0.002	0.9403	2500	0.05	0.2159
200	0.005	0.9361	3000	0.05	0.2170
