# Supplementary material for: Multi-Run Concrete Autoencoder to Identify Prognostic lncRNAs for 12 Cancers
Source: Int J Mol Sci. 2021 Nov 3;22(21):11919. doi: 10.3390/ijms222111919 (PMC8584911; doi:10.3390/ijms222111919)
Supplement: Supplementary file 1 [file ijms-22-11919-s001.zip › Supplementary/Supplementary_2_Comparison of tumor and normal features.pdf]

Supplementary 2: Comparing tumor features with normal features

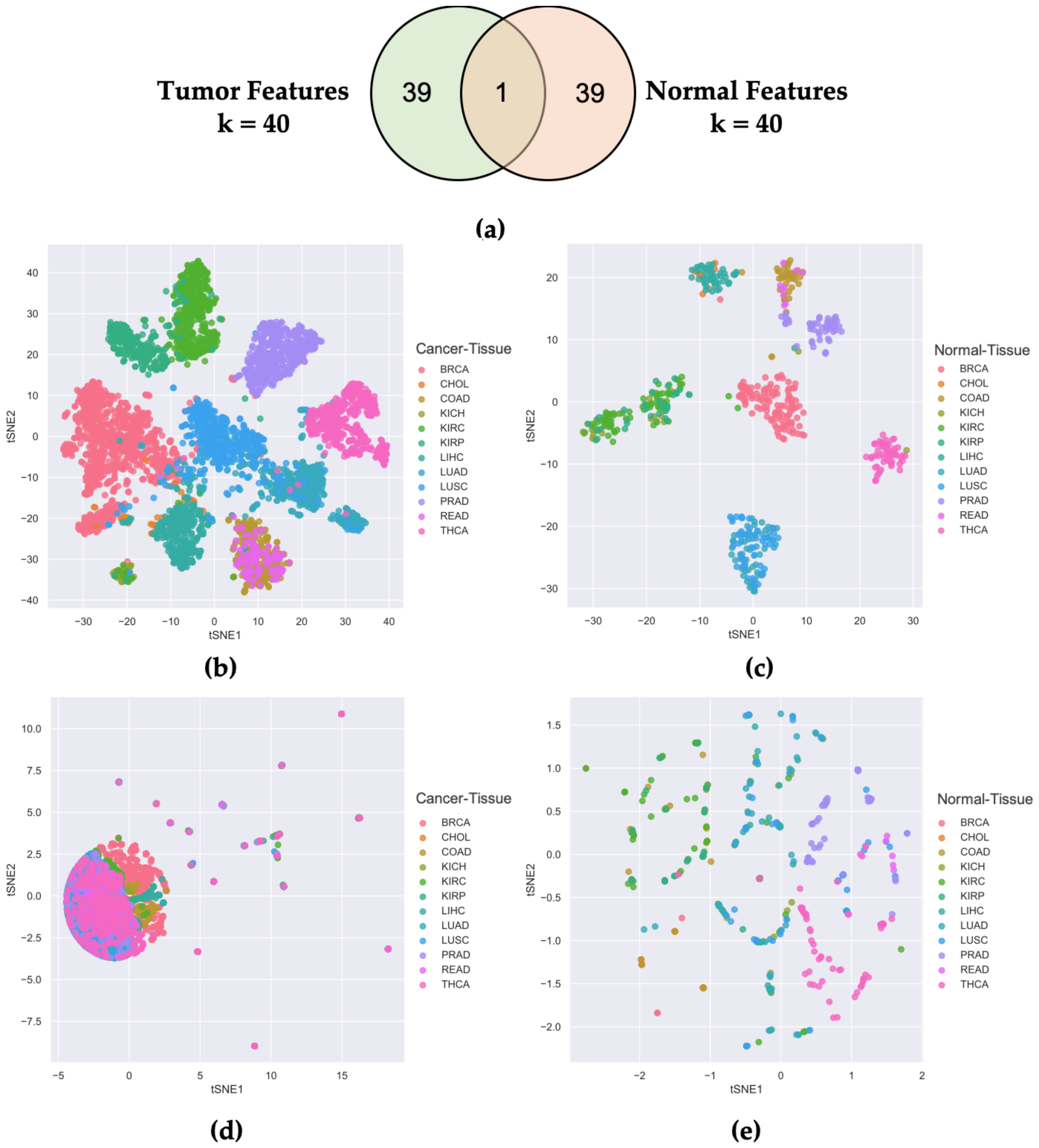

Figure 1. Comparing tumor features with normal features. a) Venn diagram of top 40 tumor features and top 40 normal features derived from CAE; b) t-SNE plot of tumor samples using tumor features; c) t-SNE plot of normal samples using normal features; d) t-SNE plot of tumor samples using normal features; e) t-SNE plot of normal samples using tumor features.

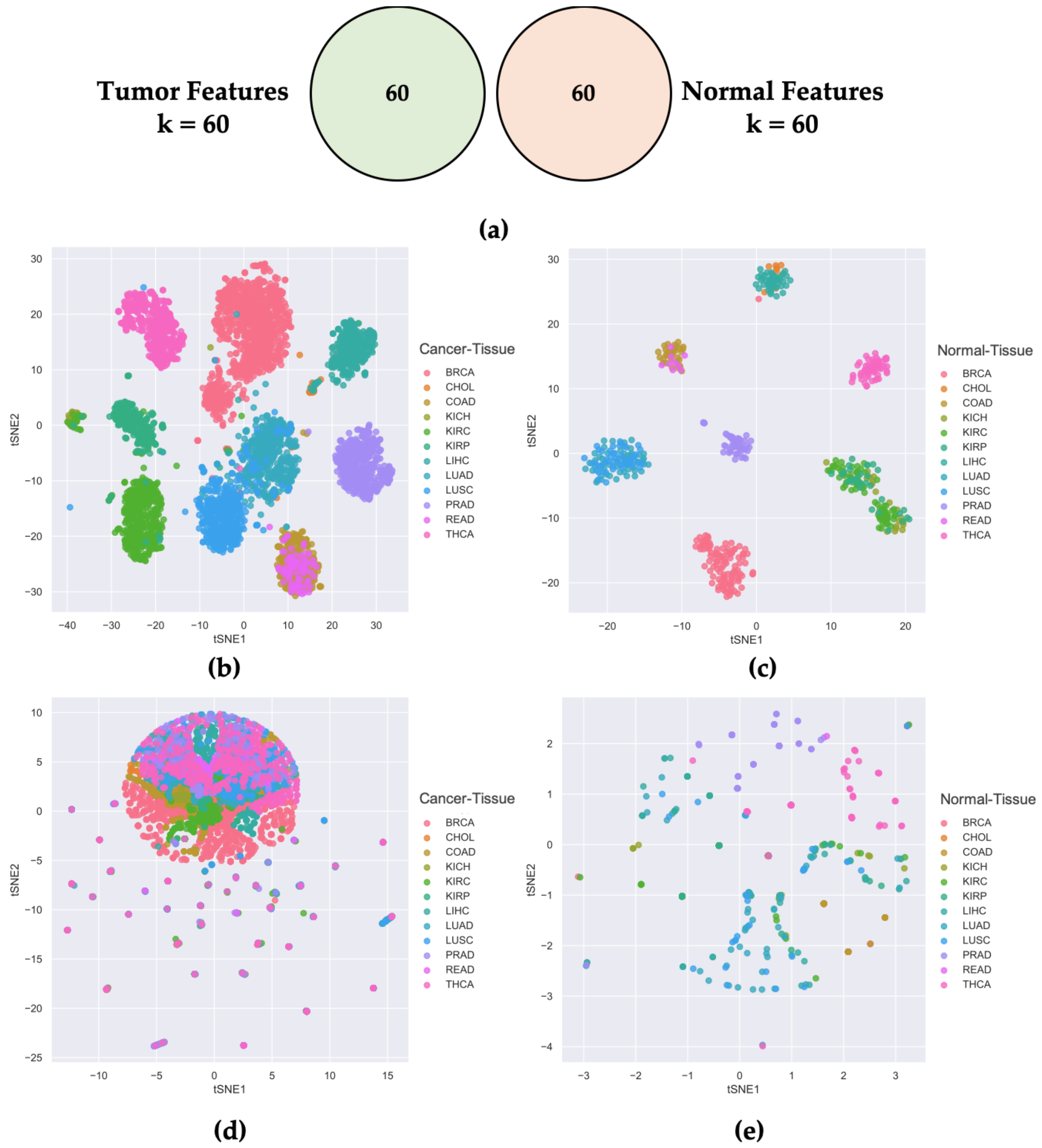

Figure 2. Comparing tumor features with normal features. a) Venn diagram of top 60 tumor features and top 60 normal features derived from CAE; b) t-SNE plot of tumor samples using tumor features; c) t-SNE plot of normal samples using normal features; d) t-SNE plot of tumor samples using normal features; e) t-SNE plot of normal samples using tumor features.

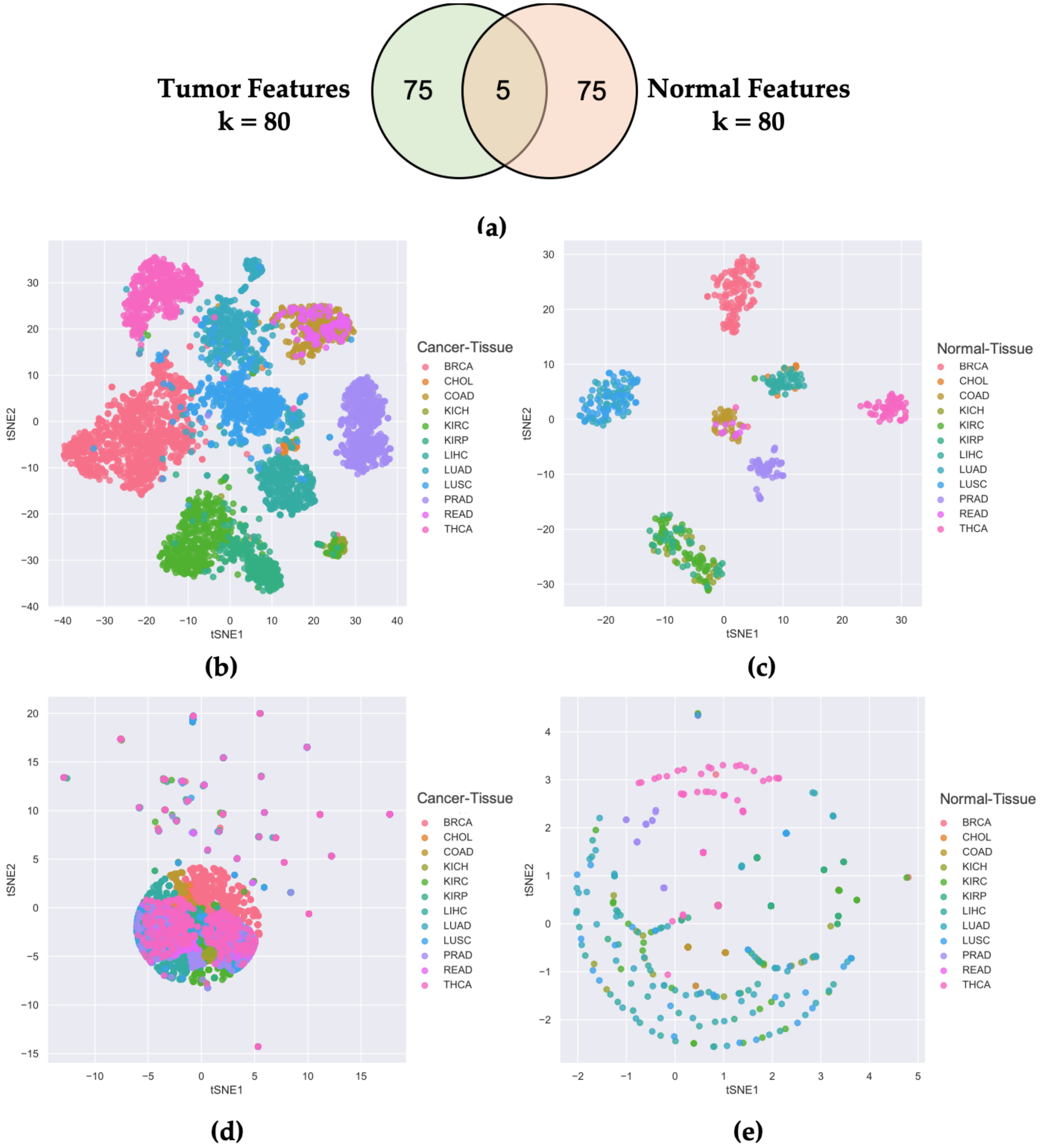

Figure 3. Comparing tumor features with normal features. a) Venn diagram of top 80 tumor features and top 80 normal features derived from CAE; b) t-SNE plot of tumor samples using tumor features; c) t-SNE plot of normal samples using normal features; d) t-SNE plot of tumor samples using normal features; e) t-SNE plot of normal samples using tumor features.
