# Supplementary material for: Multi-Run Concrete Autoencoder to Identify Prognostic lncRNAs for 12 Cancers
Source: Int J Mol Sci. 2021 Nov 3;22(21):11919. doi: 10.3390/ijms222111919 (PMC8584911; doi:10.3390/ijms222111919)
Supplement: Supplementary file 1 [file ijms-22-11919-s001.zip › Supplementary/Supplementary_4_Survival Analysis Forest Plots.pdf]

Supplementary 4: Survival Analysis (Forest Plots)

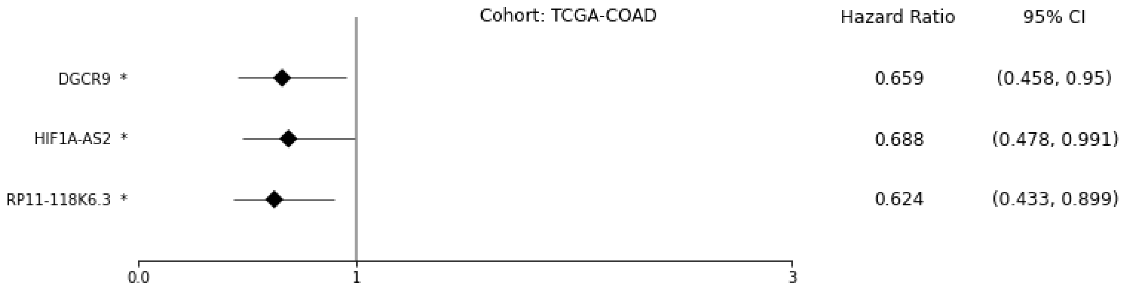

Figure 1: Forest plot of survival analysis with significant lncRNAs on TCGA-COAD cohort. The asterisks represent the Log-rank P-values: (* - P ≤ 0.05)

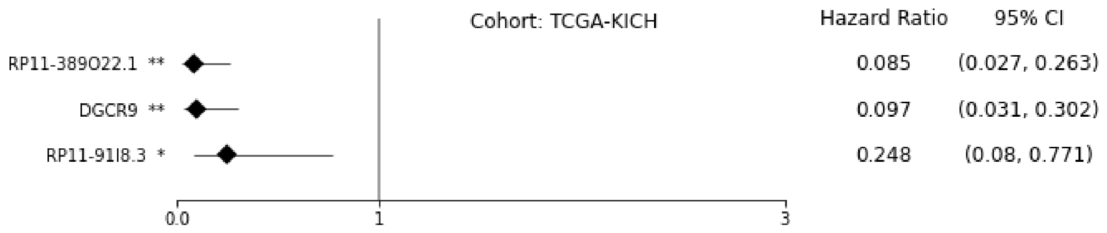

Figure 2: Forest plot of survival analysis with significant lncRNAs on TCGA-KICH cohort. The asterisks represent the Log-rank P-values: (* - P ≤ 0.05, ** - P ≤ 0.01)

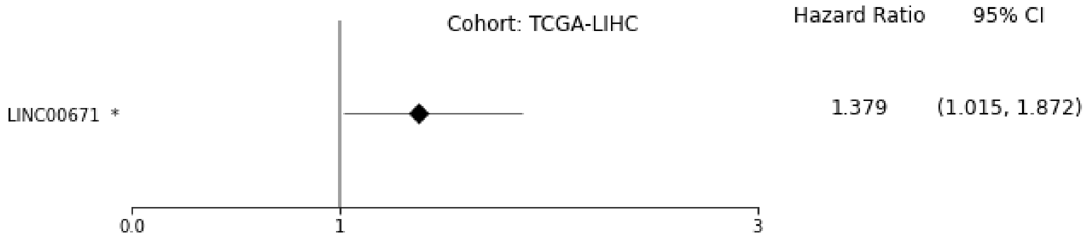

Figure 3: Forest plot of survival analysis with significant lncRNAs on TCGA-LIHC cohort. The asterisks represent the Log-rank P-values: (* - P ≤ 0.05)

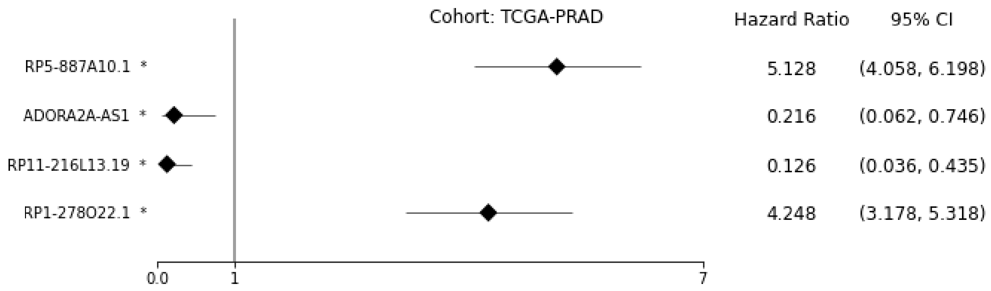

Figure 4: Forest plot of survival analysis with significant lncRNAs on TCGA-PRAD cohort. The asterisks represent the Log-rank P-values: (* - P ≤ 0.05)

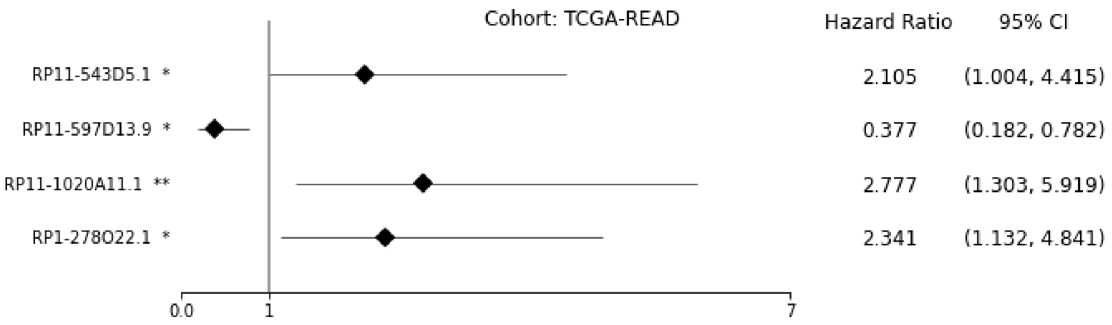

Figure 5: Forest plot of survival analysis with significant lncRNAs on TCGA-READ cohort. The asterisks represent the Log-rank P-values: (* - P ≤ 0.05, ** - P ≤ 0.01)

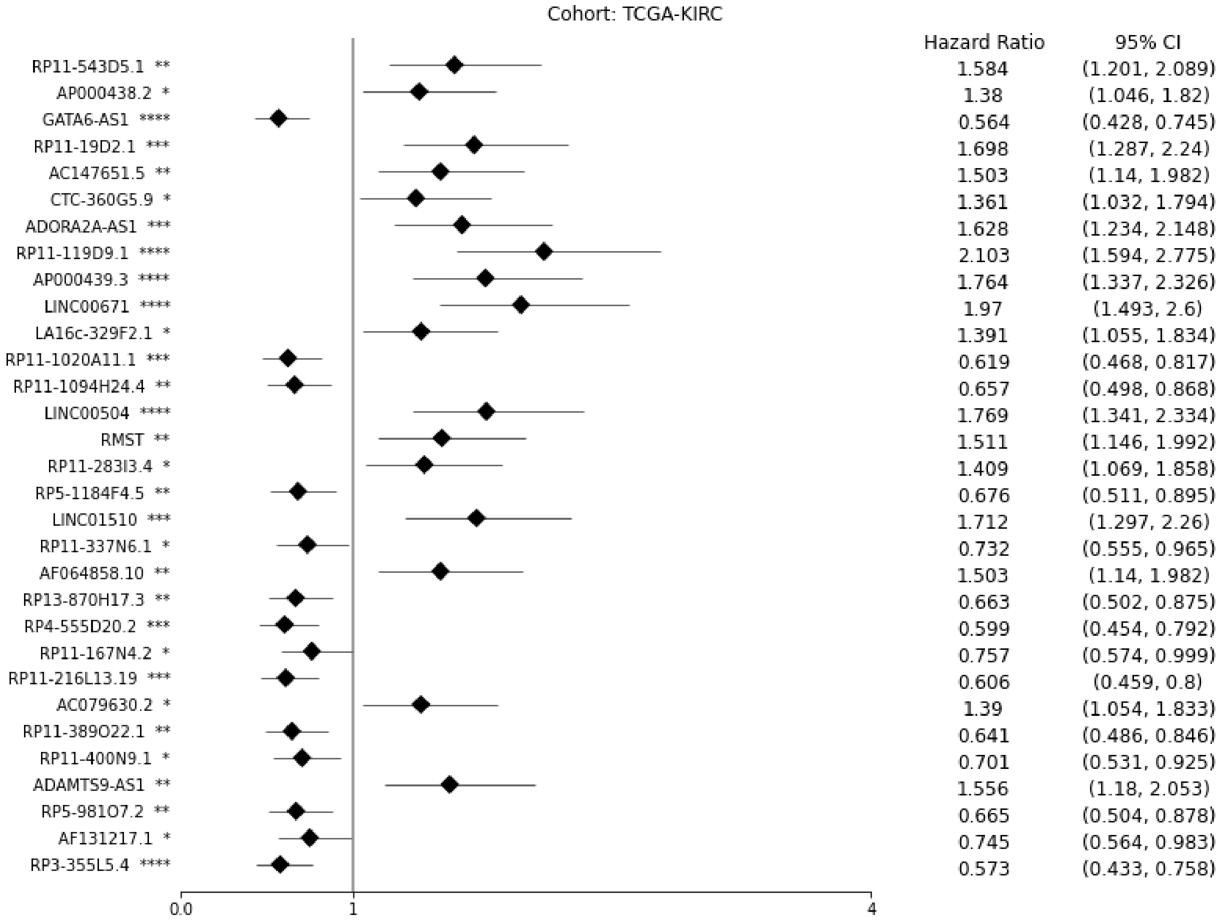

Figure 6: Forest plot of survival analysis with significant lncRNAs on TCGA-KIRC cohort. The asterisks represent the Log-rank P-values: (* - P ≤ 0.05, ** - P ≤ 0.01, *** - P ≤ 0.001, **** - P ≤ 0.0001)

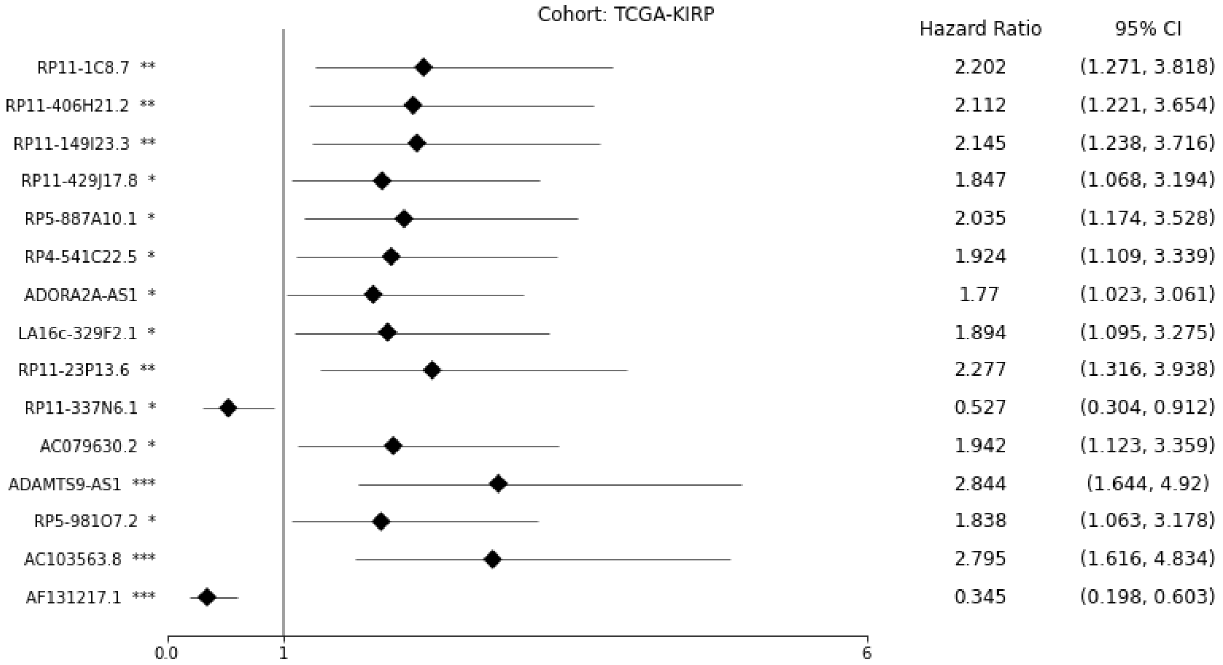

Figure 7: Forest plot of survival analysis with significant lncRNAs on TCGA-KIRP cohort. The asterisks represent the Log-rank P-values: (* - P ≤ 0.05, ** - P ≤ 0.01, *** - P ≤ 0.001)

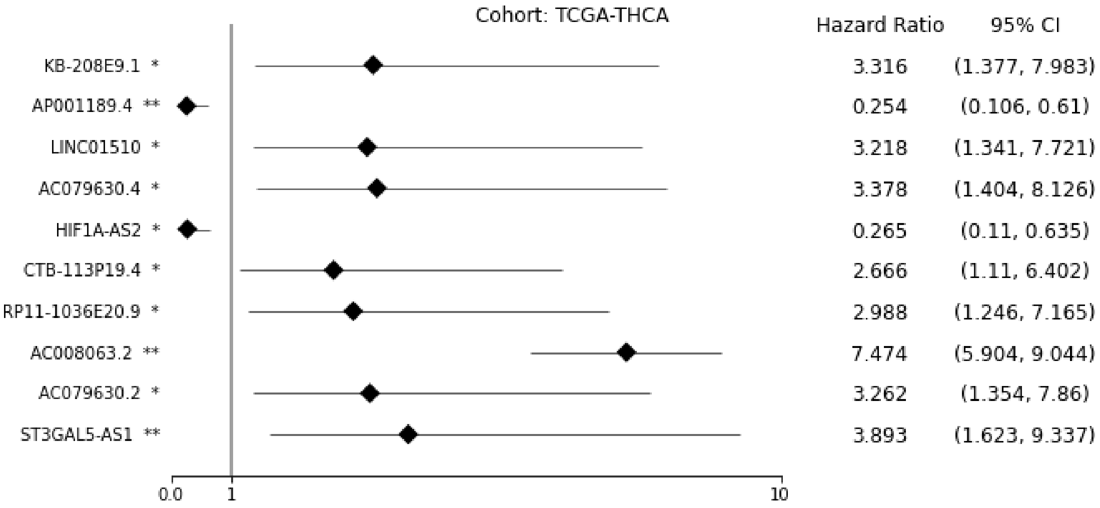

Figure 8: Forest plot of survival analysis with significant lncRNAs on TCGA-THCA cohort. The asterisks represent the Log-rank P-values: (* - P ≤ 0.05, ** - P ≤ 0.01)

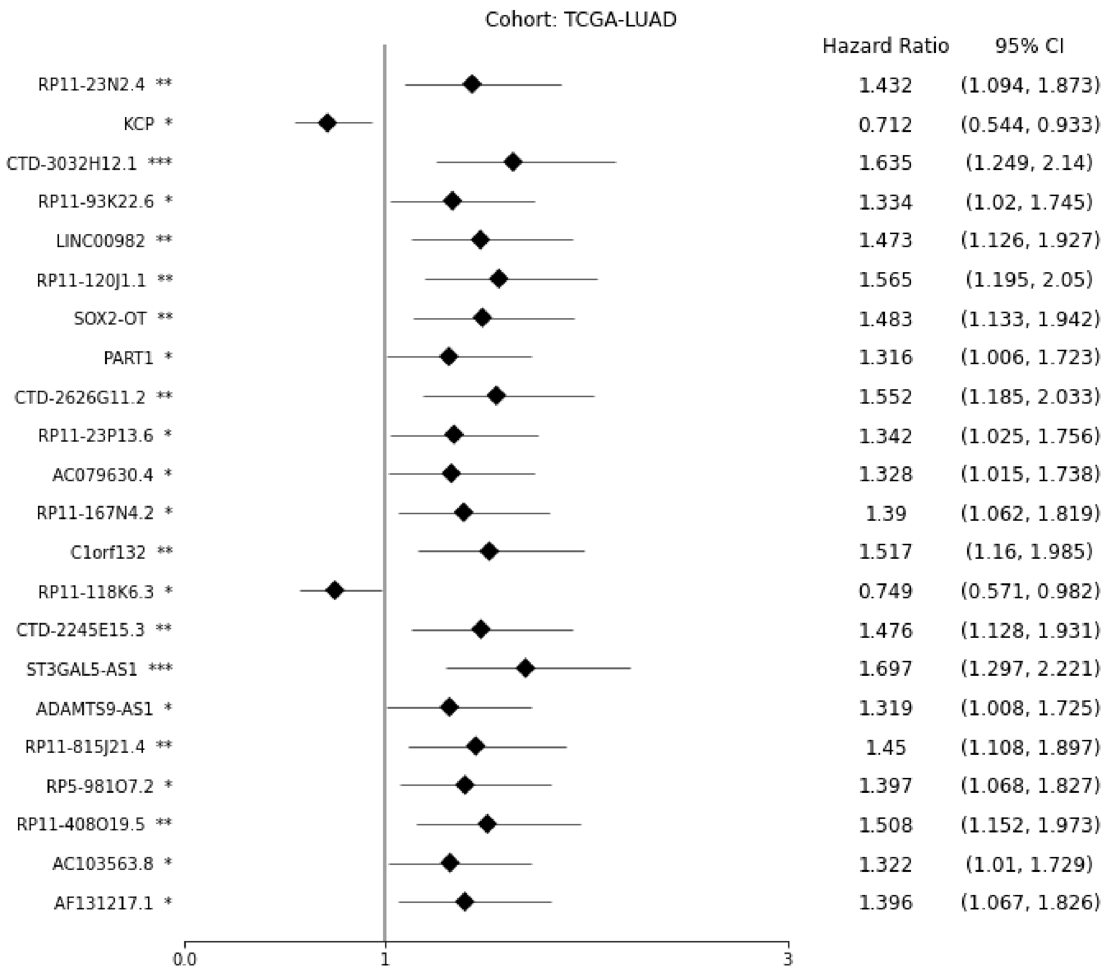

Figure 9: Forest plot of survival analysis with significant lncRNAs on TCGA-LUAD cohort. The asterisks represent the Log-rank P-values: (* - P ≤ 0.05, ** - P ≤ 0.01, *** - P ≤ 0.001)

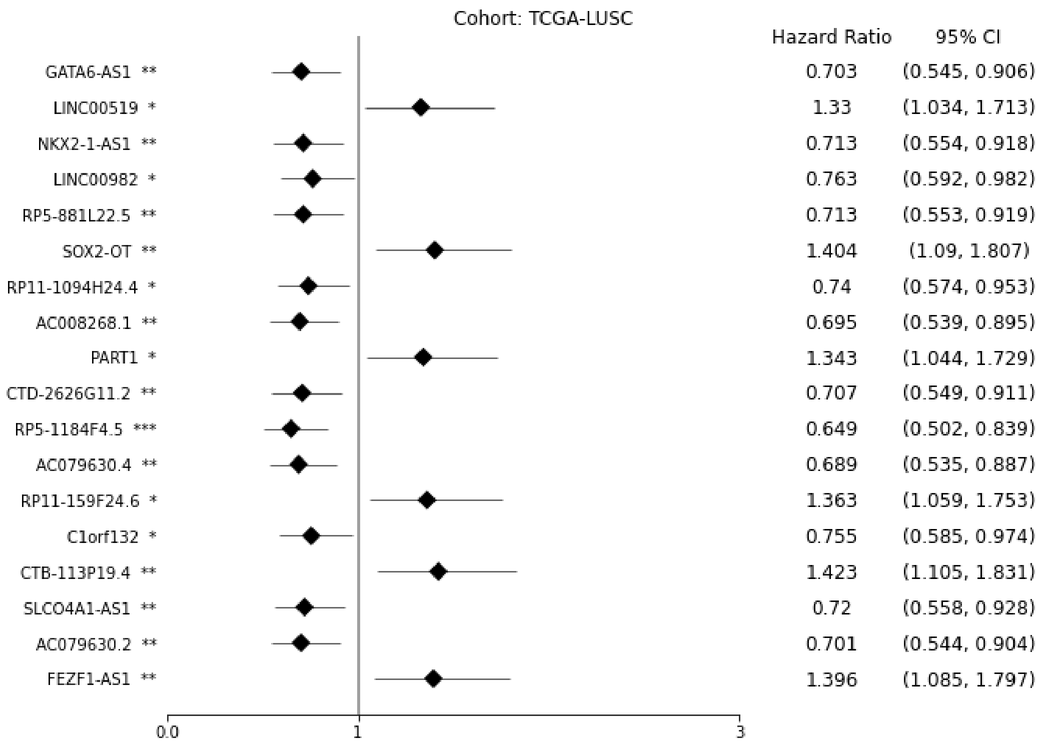

Figure 10: Forest plot of survival analysis with significant lncRNAs on TCGA-LUSC cohort. The asterisks represent the Log-rank P-values: (* - P ≤ 0.05, ** - P ≤ 0.01, *** - P ≤ 0.001)
